# ATP-binding cassette (ABC) transporters in normal and pathological lung

**DOI:** 10.1186/1465-9921-6-59

**Published:** 2005-06-20

**Authors:** Margaretha van der Deen, Elisabeth GE de Vries, Wim Timens, Rik J Scheper, Hetty Timmer-Bosscha, Dirkje S Postma

**Affiliations:** 1 University Medical Center Groningen, Department of Internal Medicine, Medical Oncology, Hanzeplein 1, 9713 GZ, Groningen, The Netherlands; 2 Department of Pulmonary Medicine, Hanzeplein 1, 9713 GZ, Groningen, The Netherlands; 3 Department of Pathology and Laboratory Medicine, Hanzeplein 1, 9713 GZ, Groningen, The Netherlands; 4 Free University, Department of Pathology, Boelelaan 1117, 1081 HV, Amsterdam, The Netherlands

## Abstract

ATP-binding cassette (ABC) transporters are a family of transmembrane proteins that can transport a wide variety of substrates across biological membranes in an energy-dependent manner. Many ABC transporters such as P-glycoprotein (P-gp), multidrug resistance-associated protein 1 (MRP1) and breast cancer resistance protein (BCRP) are highly expressed in bronchial epithelium. This review aims to give new insights in the possible functions of ABC molecules in the lung in view of their expression in different cell types. Furthermore, their role in protection against noxious compounds, e.g. air pollutants and cigarette smoke components, will be discussed as well as the (mal)function in normal and pathological lung. Several pulmonary drugs are substrates for ABC transporters and therefore, the delivery of these drugs to the site of action may be highly dependent on the presence and activity of many ABC transporters in several cell types. Three ABC transporters are known to play an important role in lung functioning. Mutations in the cystic fibrosis transmembrane conductance regulator (*CFTR*) gene can cause cystic fibrosis, and mutations in *ABCA1 *and *ABCA3 *are responsible for respectively Tangier disease and fatal surfactant deficiency. The role of altered function of ABC transporters in highly prevalent pulmonary diseases such as asthma or chronic obstructive pulmonary disease (COPD) have hardly been investigated so far. We especially focused on polymorphisms, knock-out mice models and *in vitro *results of pulmonary research. Insight in the function of ABC transporters in the lung may open new ways to facilitate treatment of lung diseases.

## Introduction

The prime role of the airways (trachea, bronchi, bronchioles and terminal bronchioles) is to conduct air into and out of the lung and to form a first line of defence against undesired constituents of inhaled air. The airways are continuously exposed to pathogens, irritants, pollutants and agents that produce oxidative stress and therefore, the composition of the respiratory tract surface is very important. The upper airways contain specialised cell types such as ciliated cells and mucous secreting goblet cells. The lower conducting airways (respiratory bronchioles, alveolar ducts and alveolar sacs) participate in gas exchange by diffusion. The alveolar epithelial surface comprises essentially two cell types, the alveolar epithelial type I cell and the cuboidal alveolar epithelial type II cell. Type I cells flatten out and in this way constitute approximately 95% of the total alveolar surface, whereas type II cells are more numerous and produce surfactant [1].

ABC (ATP-binding cassette) transporters are a family of transmembrane proteins that can transport a wide variety of substrates across biological membranes in an energy-dependent manner. They are phylogenetically classified in seven distinct subfamilies of transporters (ABCA to ABCG), which are again divided into subgroups. To date, 48 ABC transporters have been detected in the human body [2,3]. Overexpression of ABC transporters such as P-glycoprotein (P-gp) and the multidrug resistance-associated protein 1 (MRP1) were initially detected in tumour cell lines. Their overexpression is associated with increased efflux of chemotherapeutic drugs such as anthracyclines, epipodophyllotoxins and vinca-alkaloids, and this can result in so-called multidrug resistance (MDR). Many MDR proteins can act as drug efflux pumps, resulting in decreased intracellular concentrations of toxic compounds at the site of action. MRP1 and P-gp expression in the lung have especially been studied in the context of small cell lung cancer (SCLC) and non-small cell lung cancer (NSCLC). Currently we know that ABC transporters are present in virtually every cell of all species and play central roles in physiology. The prominent expression of P-gp and MRP1 in the human lung [4] suggests that these transporters may be pivotal in the protection against endogenous or exogenous toxic compounds entering the lung.The delivery of pulmonary drugs to reach the site of action may also depend on the presence and activity of many ABC transporters [5]. Langmann *et al*. developed quantitative real-time RT-PCR expression profiling of 47 ABC transporters in 20 different human tissues. Tissues with a barrier function such as lung and trachea were identified to have high transcriptional activity for many ABC transporters [6]. There are already clear proofs of important functions of ABC transporters in the lung. The ABC transporter most widely investigated is the cystic fibrosis transmembrane conductance regulator (CFTR), because mutations in this gene are responsible for the development of cystic fibrosis [7]. Mutations in *ABCA1 *are causative for Tangier disease [8]. In newborns it was shown that mutations in *ABCA3 *cause aberrant production of surfactant which can be lethal [9]. The role of altered function of ABC transporters in highly prevalent pulmonary diseases such as asthma or chronic obstructive pulmonary disease (COPD) have hardly been investigated so far [10].

This review aims to give new insights in the possible functions of ABC molecules in the lung in view of their expression in different cell types. Furthermore, their roles in protection against noxious compounds will be discussed as well as the (mal)function in normal and pathological lung. We especially focused on the members of the ABCC subfamily of transporters (to which the MRPs and CFTR belong), the ABCB subfamily (a.o. MDR1/Pgp and MDR3/Pgp) and the ABCA subfamily (a.o. ABCA1 and ABCA3), because these are a selection of the best characterised human transporters and because they have been investigated in human lungs. Results of *in vitro *studies in pulmonary research are being reviewed and an update will be given about what is known about pulmonary functional changes in ABC transporter knock-out mice models. In addition, the (potential) effects of polymorphisms in ABC transporters on lung functioning are discussed.

### MDR1/P-gp

#### MDR1 localisation and function

**Figure 1:**
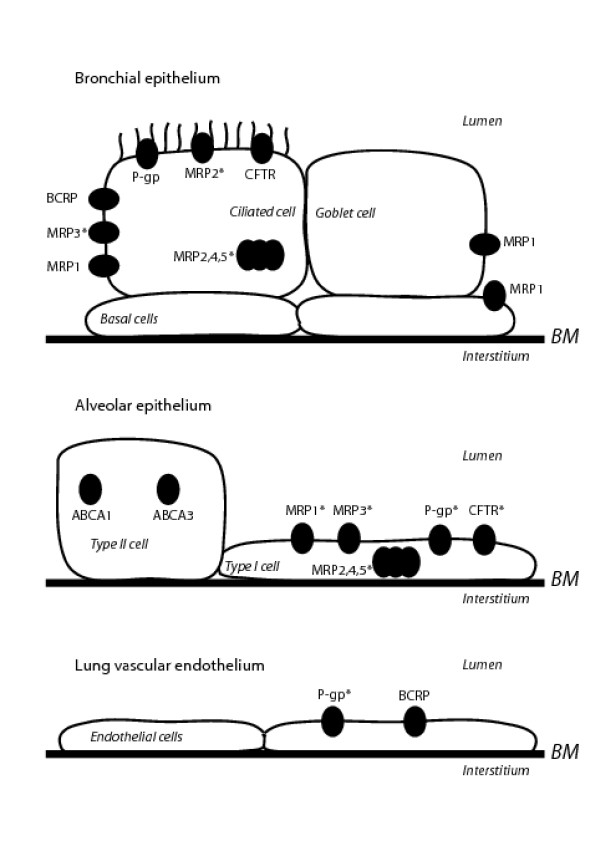
Expression of ATP-binding cassette proteins in several cell types of human lung. ABC, ATP-binding cassette; BM, basement membrane; BCRP, breast cancer resistance protein; CFTR, cystic fibrosis transmembrane conductance regulator; MRP, multidrug resistance-associated protein; P-gp, P-glycoprotein. *Conflicting results exist in literature.

**Figure 2:**
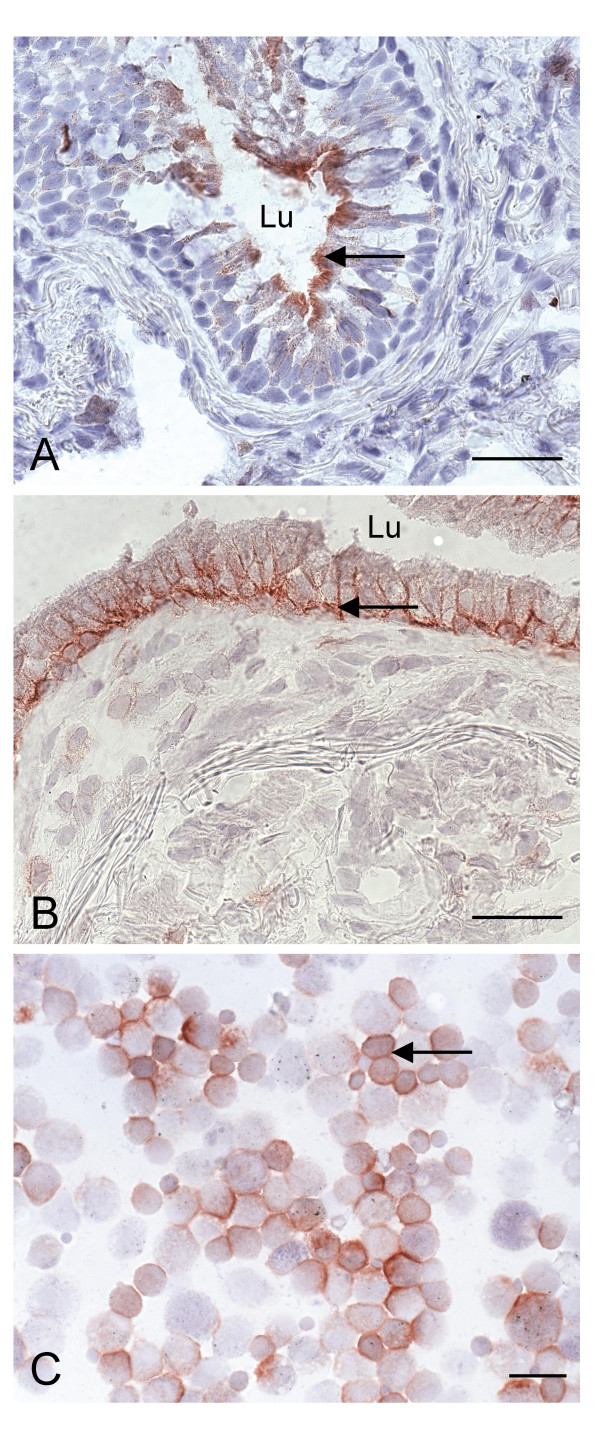
Immunohistochemical staining of ATP-binding cassette proteins in human lung. A. apical expression of P-gp in bronchial epithelium (COPD patient; frozen section, antibody C219), B. basolateral expression of MRP1 in bronchial epithelium (COPD patient; antibody MRPr1), C. MRP1 expression in bronchoalveolar lavage cells (healthy individual; antibody MRPr1). Lu, lumen. *Scale bar *= *25 μM*.

The *MDR1 *(ABCB1) gene is located on chromosome 7q21.12 and encodes for P-gp. P-gp plays a role in cell defence against environmental attacks such as generated by xenobiotics. It transports many hydrophobic substrates and anti-cancer drugs including etoposide, doxorubicin and vinblastine (for review, see [11]) and is mainly apically expressed in organs involved in excretion such as liver and intestine. In the lung, P-gp is expressed at the apical side of ciliated epithelial cells or ciliated collecting ducts, and on apical and lateral surfaces of serous cells of bronchial glands but not in mucus-secreting goblet cells (Figure 1 and 2) (Table 1) [12]. Epithelial cells of the trachea and major bronchi stain strongly while staining of the smaller bronchi is patchy or absent. In addition, P-gp is present in the lateral membranes of normal nasal respiratory mucosa [13]. In human and rat type I alveolar epithelium, P-gp is located at the lumenal side whereas freshly isolated type II cells lack P-gp [14]. In another study, pneumocytes did not stain for P-gp [15]. Some antibodies visualised P-gp in endothelial cells of blood vessels (Figure 1) [12,15,16]. In addition, alveolar and peripheral blood monocyte-derived macrophages stained positive but variably for P-gp [4,16,17]. The observed apical epithelial expression may signify that P-gp plays a role in transport of compounds from the interstitium into the lumen. However, the precise function of P-gp in the lung is as yet unknown. Interestingly, P-gp was found to play a role in the regulation of cell volume activated chloride channels and to possess channel activities [18,19]. However, Cl^- ^and K^+ ^conductances were not affected by the level of P-gp expression and the physiological role of P-gp in volume-activated chloride currents is still unclear [20-22]. Many lipophilic amines, such as fentanyl, highly accumulate in the lung. It was suggested that P-gp plays a role in the disposition of these pulmonary amine drugs [23]. Cellular uptake in lung microvascular endothelial cells of fentanyl was inhibited by the P-gp substrate verapamil, but not by the P-gp blocking antibody UIC2. The authors suggested that this results from an inhibition of fentanyl uptake. This is however unlikely, since P-gp is primarily responsible for removal of drugs out of cells.

**Table 1:** Summary of features of ABC transporters in human lung.

***ABC gene***	***Gene location***	***Functional role or substrates***	***Linked to disease***	***Protein expression in lung cells *** ***(+ localisation^$^)***	***References^#^***
MDR1/P-gp (ABCB1)	7q21.12	Drug resistance, Hydrophobic organic cations	Unknown	Bronchial epithelium (ap), mucinous glands (ap), alveolar type I cells* (ap), endothelium* (ap), alv. macroph.	[4,12,14-16]
MDR3/P-gp (ABCB4)	7q21.12	Phosphatidyl choline	Unknown	Absent	[4]
MRP1 (ABCC1)	16p13.12	Drug resistance, Organic anions (e.g. GSH conjugates, LTC4)	COPD?	Bronchial epithelium (ba/la), goblet cells (ba), peripheral epithelial cells* (ba/la), seromucinous glands (ba/la), alv. macroph.	[4,53,85]
MRP2 (ABCC2)	10q24.2	Drug resistance, Organic anions	Dubin-Johnson syndrome	Bronchial epithelium* (ap), primary bronchial and peripheral epithelial cells* (in)	[4,85,90]
MRP3 (ABCC3)	17q21.33	Drug resistance, Organic anions	Unknown	Primary bronchial and peripheral epithelial cells* (ba/la)	[85]
MRP4 (ABCC4)	13q32.1	Nucleoside analogues, Prostaglandin E_1_, E_2_	Unknown	Primary bronchial and peripheral epithelial cells* (in)	[85]
MRP5 (ABCC5)	3q27.1	Nucleoside analogues, Hyaluron	Unknown	Primary bronchial and peripheral epithelial cells* (in)	[85]
MRP6 (ABCC6)	16p13.12	Unknown	Pseudoxanthoma elasticum	Unknown	
MRP7 (ABCC10)	6p21.1	Drug resistance	Unknown	Unknown	
MRP8 (ABCC11)	16q12.1	Conjugated steroids, Nucleoside analogues, bile acids	Unknown	Unknown	
MRP9 (ABCC12)	16q12.1	Unknown	Unknown	Unknown	
CFTR (ABCC7)	7q31.31	Chloride ion channel	Cystic fibrosis	Bronchial epithelium (ap), seromucinous glands (ap), Clara cells*, Alv. type I cells*	[117-120]
BCRP (ABCG2)	4q22	Drug resistance, Protection food toxins	Unknown	Bronchial epithelium (ba/la), endothelium, seromucinous glands	[4]
ABCA1	9q31.1	Cholesterol and phospholipids	Tangier disease	Alv. type II cells	[151]
ABCA3	16p13.3	Surfactant secretion	Surfactant deficiency	Alv. type II cells	[155]

*^$^Cellular localisation: ap, apical; ba, basal; la, lateral; in, intracellular; alv., alveolar; macroph., macrophages; ^#^References are mentioned that demonstrate protein localisation histologically;* *Conflicting results exist in literature.

P-gp mRNA expression was not increased in smokers (n = 11) compared to ex-and non-smokers (n = 7) [12]. Whether P-gp expression levels may play a defensive role towards tobacco-derived agents remains to be investigated.

#### MDR1 in tumours

High P-gp expression can imply chemotherapeutic resistance due to increased chemotherapeutic drug efflux. In cancer therapy, many attempts have been made to reverse MDR mechanisms. However, in a randomised double-blind trial in 130 SCLC patients no positive effects were seen with the P-gp modulator megestrol acetate in addition to chemotherapeutic drugs, suggesting that levels of P-gp expression in lung tumours were not relevant or that modulation of P-gp activity was not complete in this treatment [24]. Some studies show higher P-gp expression at the invasion front of lung tumours and it was suggested that P-gp expression augments invasion properties of tumour cells [25]. Only two out of 22 NSCLC samples (both adenocarcinomas) stained positive with three P-gp antibodies [15] and no P-gp was detected on pulmonary carcinoids. Other studies revealed a relation between P-gp and glutathione S-transferase-pi (GST-pi) expression in NSCLC that were exposed *in vitro *to doxorubicin [26], suggesting that these two factors play a role in doxorubicin resistance. There was also a correlation between current smoking and doxorubicin resistance of NSCLC. Forty-two out of 72 NSCLC smokers expressed P-gp, whereas only two out of 22 tumours of non-smokers were P-gp positive [27].

#### MDR1 polymorphisms

*MDR1 *polymorphisms were first described by Hoffmeyer *et al*. [28] who found a correlation between lower intestinal expression of P-gp and a polymorphism in exon 26. Many single nucleotide polymorphisms (SNPs) have been recognised in the *MDR1 *gene (see reference [29] for recent review about clinical aspects). The impact of these polymorphisms on lung diseases is still speculative. It was proposed that polymorphisms in the *MDR1 *gene may have clinical consequences in patients with cystic fibrosis, since MDR1 plays a role in CFTR regulation. Rodents contain two *Mdr1 *genes, denoted as *Mdr1a *and *Mdr1b*. It was shown that Mdr1b mRNA expression in lung parenchyma of outbred rats is very variable and this may also be the case in humans [30]. The possible effects of *MDR1 *polymorphisms was studied in tobacco-related lung cancer [31]. No clear association was found between the T/T genotype of the C3435T polymorphism and susceptibility to lung cancer in a group of 268 Caucasian men who were current smokers. No relation was found between SNP C3435T in *MDR1 *and survival in 62 docetaxel-cisplatin-treated NSCLC patients [32]. Immunosuppressive agents such as cyclosporin A and tacrolimus (both calcineurin antagonists) are P-gp substrates. No relation was found of *MDR1 *G2677T and C3435T genotypes with tacrolimus blood levels in 83 lung transplant patients treated with tacrolimus [33]. Altogether, these data implicate that there is still no clear association between *MDR1 *polymorphisms and effects on outcome of treatment of lung cancer or lung transplant patients.

#### MDR1 in animal models

Scheffer *et al*. detected high P-gp levels in lungs of mice [4]. In rats, Mdr1a and Mdr1b mRNA expression were highest in the ileum [34]. The Mdr1a expression level in rat lung was 2% of the expression in ileum and expression of Mdr1b was 47% of that in ileum. In mice orally treated with dexamethasone for 24 hours, Mdr1b mRNA expression in lungs was decreased, from which the authors deduce that dexamethasone treatment of lung tumours may reverse MDR [35]. To study the *in vivo *distribution of P-gp, nude rats were injected with a P-gp overexpressing SCLC cell line (GLC4/Pgp) and with a P-gp negative cell line (GLC4) [36]. P-gp function was visualised with radiolabeled P-gp substrate [^11^C]verapamil by positron emission tomography (PET) with or without P-gp modulator cyclosporin A. The accumulation of [^11^C]verapamil was significantly increased by cyclosporin A in brains and GLC4/Pgp tumours in these rats. In all other investigated organs including lungs, the accumulation after cyclosporin A treatment was unaltered. In intact rabbit lung, vascular P-gp kinetics were measured in vivo using the lipophilic amine dye rhodamine 6G (R6G) by measuring R6G in the perfusate during circulation [37]. Inhibition of P-gp function with verapamil or GF120918 resulted in higher accumulation of R6G in lung. It was proposed that the opposite would happen when epithelial P-gp was inhibited because R6G would then be retained in the airspace. We propose another possibility that inhibition of epithelial P-gp will also result in higher R6G accumulation. In that case, R6G transport to the lumen is inhibited and as a compensation mechanism it may be transported back to the interstitial side where it either may be retained in the tissue or subsequently be transported into the circulation. This model could be useful in testing a large variety of pulmonary therapeutic agents, such as corticosteroids and sympathicomimetics that may be substrates for transporters in the lung or modulate their activity. Similar studies were also carried out in perfused rat liver to assess the effect of P-gp modulators on the hepatobiliary system, supporting the usefulness of this approach [38].

Mdr1a/1b (-/-) mice lack both genes encoding for P-gp and these mice seem physiologically normal. The penetration of [^3^H]digoxin (a P-gp substrate) was higher in brain, ovary and adrenal gland. In the lung tissue of these (-/-) mice [39], the level of [^3^H]digoxin was rather low compared to other organs. The level was 2.6 times higher in (-/-) mice than in (+/+) mice, but this was not significant. The pharmacokinetics of the central nervous system (CNS) drug amitryptiline (a P-gp substrate) and its metabolites were examined in Mdr1a/1b (-/-) mice by high performance liquid chromatography (HPLC) in several organs. Higher concentrations of these metabolites were measured in the brain of these mice, but not in other examined organs including the lung [40]. Thus, P-gp likely plays a more active role in exporting CNS drugs out of the brain than out of the lung. Another possibility is that other transporters in the lung were capable to efflux these drugs as a compensation mechanism.

#### MDR1 in vitro

Several lung cell culture models have been described for determining P-gp expression and functionality. The immortalised human bronchial epithelial cell line 16HBE14o^- ^resembles primary epithelium and was reported to be suitable for drug metabolism studies [41]. In 16HBE14o^- ^cells, P-gp was expressed at the apical side. Its functional activity was measured with P-gp substrate rhodamine 123 and its transport was inhibited by verapamil [42]. Two lung cell lines, Calu-3 and A549 cells, were compared for P-gp expression and functionality [43]. The bronchiolar adenocarcinoma cell line Calu-3 is suitable for drug transport studies because these cells form tight junctions in culture, which is not the case for the type II alveolar carcinoma cell line A549. P-gp expression was higher in A549 cells than in Calu-3 cells. However, efflux of rhodamine 123 was higher in Calu-3 cells. This may be explained by additional MRP1 activity in these cells because rhodamine 123 is also an MRP1 substrate [44]. In primary rat alveolar type II cells, Mdr1b mRNA levels increased in a time dependent manner in cultures at day 1, 2 and 3 compared to freshly isolated cells. Mdr1b mRNA was present at low levels and increased after oxygen radical induction with paraquat [45]. P-gp expression was below the detection limit in these cells at time of isolation. In freshly isolated primary human bronchial epithelial cells, P-gp was present and increased after 24 hours paraquat exposure. Rhodamine 123 efflux could be measured in these cells, confirming functional activity of P-gp [45]. These results demonstrate that P-gp is upregulated during stress, both from radical production and from *ex vivo *culturing or differentiation.

### MDR3/P-gp

The MDR3/P-gp (ABCB4) gene maps closely to MDR1/P-gp on chromosome 7q21.12 and has high homology with MDR1 although its function is very different. It is involved in phosphatidyl choline transport from the liver into the bile. Its RNA and protein have not been detected in human and mouse lung and trachea [4,6]. and it is therefore not expected that MDR3 is present in the lungs.

### MRP1

#### MRP1 localisation and function

The *MRP1 *(ABCC1) gene is located on chromosome 16p13.12. Cole *et al*. discovered in 1992 a non-Pgp mediated MDR mechanism in the human lung cancer cell line H69AR [46]. MRP1 was overexpressed in these cells and it was found that glutathione-, glucuronide-, and sulfate-conjugated organic anions are substrates for MRP1 [47,48]. MRP1 confers resistance to several chemotherapeutic agents including vincristine, daunorubicin and methotrexate [49-51]. Physiological substrates for MRP1 are e.g. leukotriene C_4 _(LTC_4_) and glutathione disulfide (GSSG) [52]. Interestingly, these substrates play an important role in lung physiology with respect to inflammation and oxidative stress. MRP1 is highly expressed mainly at the basolateral side of human bronchial epithelial cells (Figure 1 and 2) [53]. Ciliated and basal cells have been collected from brushes of main or lobar bronchi. Basal cells stained strongly on the entire circumference of the plasma membrane. Ciliated epithelial cells and mucous cells stained positive at the basolateral membrane but not at the apical membrane. No intracytoplasmic staining was observed. However, strong apical cytoplasmic staining has been detected below the cilia in respiratory columnar epithelial cells in paraffin sections with antibody MRPr1 [4,13,54]. The discrepancy between these findings may be due to different fixation procedures. Basal cells of seromucinous glands of the lungs also stain positive for MRP1 with a higher intensity in the serous area than mucinous cells. Alveolar macrophages are MRP1 positive with variable staining between individual lung samples (Figure 2) [4]. Kool *et al*. investigated MRP1, 2, 3, 4, and 5 with an RNase protection assay in total lung RNA and found high MRP1, absence of MRP2, low MRP3 and MRP4 and moderate MRP5 gene transcripts [55]. Total RNA was obtained from lung tissue collected during surgery or autopsy. Therefore, the original cell types can not be distinguished in these samples.

Possibly, the high MRP1 expression at the basolateral side of lung epithelium may assist in the clearance of toxins coming from the luminal or interstitial side (back) into the interstitial fluid [56]. Function of MRP1 expression in the lung epithelium, glands and alveolar macrophages may include extrusion of toxic intracellular substances, antioxidant defence or production of LTC_4 _as an inflammatory response. Given the fact that MRP1 expression is higher in the lung compared to other solid organs, decreased or increased functional MRP1 expression may have a high impact on development and/or progression of lung diseases and protection against air pollution and inhaled toxic substances such as present in cigarette smoke [10].

#### MRP1 in tumours

Using mRNA *in situ *hybridisation Thomas *et al*. [57] detected high MRP1 expression in normal epithelium whereas the major component of the tumour epithelium showed a negative hybridisation signal. However, MRP1 transcript expression at the invasive front of the lung tumours was consistently stronger and particularly strong in areas with lymphatic or blood vessels. In addition, endothelial cells and lymphocytic infiltrates stained strongly positive for MRP1. These results may indicate a role of MRP1 in invasion or in mitotic activity. In a recent study, all 102 NSCLC tumours were MRP1 positive. In addition, the level of expression was 3-fold higher in DNA-aneuploid cells compared to normal bronchial and carcinomatous DNA-diploid cells [58]. This was associated with more frequent gain of chromosome 16 where the *MRP1 *gene is located. Overexpression of MRP1 may therefore be an important factor of intrinsic resistance to chemotherapy in NSCLC. High MRP1 expression can also result in increased apoptosis as shown by experiments with verapamil in *MRP1 *transfected baby hamster kidney-21 (BHK-21) cells [59]. Verapamil acts as an apoptogen in these cells when compared to MRP1 negative cells or mutant *MRP1 *transfected control cells. This was accompanied by depletion of intracellular GSH due to transport of GSH by MRP1. Indeed, addition of extracellular GSH prevented cell death. This mechanism may be valuable for treatment of MRP1-positive tumours.

#### MRP1 in non-malignant diseases

Cigarette smoking is the principle risk factor for the development of COPD. Our preliminary results indicate that MRP1 expression is diminished in bronchial epithelium of COPD patients (ex-smokers) and that lower expression is related to worse lung function [10]. In addition, bronchial MRP1 expression was higher during smoking than after one year of smoking cessation (Van der Deen *et al*., submitted). The expression of MRP1 measured with RT-PCR was similar in lungs of smokers and a combined group of ex- and non-smokers, suggesting that current smoking does not affect MRP1 gene expression [53]. This was semi-quantitatively analysed and the power of this study was rather low (smokers n = 13 and ex- or non-smokers n = 8). Cigarette smoke extract is a complex mixture of many substances. One of these is nitrosamine 4-(methylnitrosamino)-1-(3-pyridyl)-1-butanone (NNK), a carcinogenic nitrosamine. NNK is converted intracellularly in NNAL-*O*-glucuronide which is transported by MRP1 in the presence of glutathione (GSH) [60]. Thus, functional activity of MRP1 in the lung may play an important role in the antioxidant defence against toxic compounds generated by cigarette smoke.

#### MRP1 polymorphisms

Polymorphisms of the *MRP1 *gene or in regulatory genes may influence function of MRP1 in the lung. *MRP1 *has been screened for genetic variations and several mutations have been found in the *MRP1 *gene in the human population [61,62]. Ethnic differences for MRP1 expression were observed between Caucasian and Japanese subjects but the clinical consequences have to be determined to date [63,64]. In preclinical models, a single mutation in the *MRP1 *gene can result in loss of transport of some, but not all, MRP1 substrates [65] which implies that potentially minor differences in the *MRP1 *gene may result in aberrant functional capacities of the MRP1 protein. A low frequency (<1%) naturally occurring mutation in *MRP1 *was related to functional differences in organic anion transport and drug resistance [66]. Therefore, some individuals could be more susceptible to a set of xenobiotics than others.

#### MRP1 in animal models

Scheffer *et al*. detected, besides P-gp, high levels of Mrp1 in lungs of mice [4]. *Mrp1 *(-/-) mice develop normally [67]. Viability, fertility, and a range of histological, hematological, and serum-chemical parameters were similar in *Mrp (+/+) *and *Mrp (-/-) *mice. However, *Mrp1 (-/-) *mice are hypersensitive to exposure to several drugs e.g. etoposide, resulting in loss of body weight and death [56,67,68]. In Mrp1 deficient mice there were no gross abnormalities in lungs and other tissues after treatment with the chemotherapeutic drug etoposide-phosphate, but abnormal mucous production was seen around the mouth. Further examination of tissues showed that these mice suffered from oropharyngeal toxicity [56]. In *Mrp1 (-/-) *mice, GSH levels were elevated in e.g. lung, kidney, heart, testes and skeletal muscle. In organs that express little MRP1, such as the liver and small intestine, GSH levels were unchanged [67,69]. The increase in GSH was not related to increased levels of gamma-glutamylcysteine synthetase (γ-GCS), the rate-limiting enzyme for production of the tripeptide GSH. This suggests that MRP1 plays a role in GSH metabolism because MRP1 expression determines cellular GSH levels which is γ-GCS independent. In liver tissue, Mrp2 and Mrp5 mRNA levels were increased in *Mrp1(-/-) *mice compared to wild-type mice, probably due to a compensation mechanism. Mdr1a and Mrp3 levels were unchanged in these animals. Unfortunately, lung tissue was not examined [69]. *Mrp1 (-/-) *mice showed impaired inflammatory responses accompanied by a decreased LTC_4 _excretion by leukotriene producing cells [67,68]. The outgrowth of *Mycobacterium tuberculosis *was enhanced in *Mrp1 (-/-) *mice, but there was no difference in survival compared to wild-type mice [70]. Strikingly, Schultz *et al*. [71] observed that the survival of *Mrp1 (-/-) *mice inoculated with *Streptococcus pneumoniae *was better than of wild-type mice. This was accompanied by a lower LTC_4 _concentration but a higher LTB_4 _level in bronchoalveolar lavage fluid (BALF). Treatment with an LTB_4 _antagonist abolished the positive effect on survival rate. Altering MRP1 function may therefore be a target for further studies on treatment of pneumonia. Interestingly, LTB_4 _levels are also elevated in sputum and exhaled breath condensate of COPD patients [72,73]. This is thought to result from a higher amount of neutrophils present, yet also lower functional MRP1 expression may play a role, according to the observations in *Mrp1 (-/-) *mice [10,71]. The pulmonary and hepatic carcinogen aflatoxin B_1 _(a mycotoxin) and its GSH conjugate are MRP1 substrates *in vitro *[74]. MRP1 may therefore play an important role in detoxification of aflatoxin B_1 _in the lung. No difference in occurrence of lung tumours (and liver tumours) 12 months after an 8 week exposure to aflatoxin B_1 _was observed in *Mrp1 (-/-) *and wild-type mice [75]. This observation may be explained by differences in exposure time compared to humans (who may be chronically exposed for many years) or by redundancy of other ABC transporters in knock-out mice to export aflatoxin B_1 _and its conjugates.

NRF2 (nuclear factor-E2 p45-related factor), a transcription factor for many genes that play a role in antioxidant defence and detoxification processes, was recently identified as a transcription factor for MRP1 [76]. Interestingly, the onset of cigarette smoke-induced emphysema was earlier and the extent of emphysema was more severe in *Nrf2 *(-/-) mice as compared to wild-type mice [77]. The number of inflammatory cells, mainly consisting of macrophages, in bronchoalveolar lavage fluid and lung tissue was higher in *Nrf2 *(-/-) mice, accompanied by more extensive apoptosis of endothelial cells and alveolar type II cells. Forty-five Nrf2-dependent protective genes were induced by smoke exposure in wild-type mice compared to knock-out mice as measured with microarray analysis. Regretfully, *Mrp1 *was not present on this gene expression array. Similar smoke exposure experiments with *Mrp1 *(-/-) mice would be interesting to investigate if these mice are also more susceptible to develop emphysema. Three polymorphisms were identified in the promotor region of *NRF2 *in healthy individuals (n = 81) and in patients with COPD (n = 87) and sytemic lupus erythematosis(n = 51) (all Japanese individuals) [78]. There was no relation between these polymorphisms and risk of these diseases, but the power of this study was rather low.

*Mdr1a/1b(-/-)/Mrp1(-/-) *triple knockout (TKO) mice are indistinguishable from wild-type mice under normal physiological conditions [79,80]. Intravenous injection of [^3^H]etoposide in TKO mice resulted after 4 hours in a higher accumulation in brown adipose tissue, colon, urogenital tract, salivery gland and heart when compared to *Mdr1a/1b (-/-) *mice. The accumulation of [^3^H]etoposide in lung did not increase [79]. Accumulation of [^3^H]vincristine was 26-fold higher in lungs of weaning TKO mice compared to adult TKO mice, 4 hours after a single dose intraperitoneal injection [81]. In TKO compared to wild-type mice, [^3^H]vincristine was 82.7 and 5.8-fold increased in respectively weaning and adult mice after 4 hours in this experimental setting. Eight hours post-injection, these ratios were 33 and 51.1. Thus, accumulation of vincristine is both time and age-related. In another study, toxicity of the chemotherapeutic drugs vincristine and etoposide was elevated in TKO mice (respectively 128-fold and 3–5 fold) compared to wild-type mice. Lung tissue was not further examined [80].

#### MRP1 in vitro

MRP1 overexpression was first described in the human lung cancer cell line H69AR [46]. The human SCLC cell line GLC4/ADR, which displays multiple copies of the *MRP1 *gene, is often used to study MRP1 function [47]. The lung cell line Calu-3 (from bronchiolar adenocarcinoma/glandular origin) has been reported to express MRP1 basolaterally and has functional MRP1 activity. It may serve as a good model for *in vitro *analysis of transport activities [44]. Besides P-gp, MRP1 protein was present in freshly obtained primary human bronchial epithelial cells. Efflux of MRP1 substrate carboxy-dichlorofluorescein could be measured in these cells and was inhibited by MRP modulator MK571 [45]. In contrast to upregulation of P-gp, epithelial cells did not respond with MRP1 upregulation to paraquat and MRP1 levels were stable in time until 12 weeks, but highly increased after 18 weeks culturing. An interesting observation was made by Bandi *et al*. [82] who incubated the airway epithelial cell line Calu-1 with budesonide, an anti-asthma drug. MRP1 expression decreased after 7 and 14 days incubation with budesonide (10 μM) but not at day 1 and 4. MRP1 function was also diminished after 14 days incubation with increasing amounts of budesonide (1, 10 and 100 μM). It was therefore proposed that budesonide could be used as a chemosensitiser in lung tumours. The immortalised bronchial epithelial cell line 16HBE14o^- ^expresses high MRP1 protein levels and activity [83]. MRP1 activity, but not P-gp, was blocked by incubation with cigarette smoke extract. Thus, the expression of MRP1 in human lungs may contribute to defence mechanisms of toxic inhaled substances present in cigarette smoke to protect against pulmonary diseases such as lung cancer or COPD. It was shown that benzo [a]pyrene (B [a]P), a polycyclic aromatic hydrocarbon that is a.o. present in cigarette smoke and car exhaust fuels, increased the efflux of monochlorobimane GSH conjugate (mBCl-SG, which is an MRP substrate) out of primary rabbit alveolar type II cells [84]. This was explained by an increase in GSH levels. MK571 reduced the efflux of mBCL-SG, suggesting that the transport was MRP-mediated. Surprisingly, the transport direction of this substrate was higher to the apical than to the basolateral compartment. This may imply that MRP1 (or other MRPs) is apically located in alveolar type II cells in contrast to the basolateral location that is usually reported for MRP1, or that another transporter is responsible for this unexpected result. MRP1-5 expression was studied in primary human lung cell cultures and in A549 cells [85]. MRP1 and MRP3 expression were membrane-associated whereas MRP2, 4, and 5 were located in intracellular structures in primary cells (both bronchial and peripheral epithelial cells). In A549 cells, all transporters were expressed in the cellular membrane. The fluorescent microscopic pictures, however, did not precisely show the exact cellular localisation and in several cases, the Golgi apparatus also seems to stain beside the cellular membrane.

### MRP2

#### MRP2 localisation and function

*MRP2 *(ABCC2) is located on chromosome 10q24.2 and is also named the canalicular multispecific organic anion transporter (cMOAT) in the liver [86]. MRP2 and MRP1 share very similar substrate specificities. High affinity endogenous substrates for MRP2 include amphiphilic anions, such as LTC_4 _[87,88]. and bilirubin glucuronosides [89,90]. Data on the presence of MRP2 protein in the lung are conflicting. Only one out of three antibodies stained positive at the apical side in bronchial epithelium (Figure 1) [4,90]. If MRP2 is expressed apically, it could be involved in transport of noxious compounds into the lumen of the lung.

#### MRP2 in animal models

There are several rat strains that have a mutation in *Mrp2/cMOAT *and the phenotypes resemble that of the human Dubin-Johnson syndrome, a disease in which *MRP2 *is mutated. Examples are the GY/TR^- ^rat [91] and the Eisai hyperbilirubinemic rat (EHBR) [92]. Subjects with the Dubin-Johnson syndrome and also *Mrp2 *deficient rats display jaundice and have impaired organic anion transport. Recently, it was shown that deficient Mrp2 function in EHBR rats was compensated by upregulation of Mrp3 in liver and kidney [93]. *Mrp2 (-/-) *mice have not been described. There are no reports in the literature of pulmonary malfunction in rats or humans with defects in the *MRP2 *gene.

### MRP3

The *MRP3 *(ABCC3) gene is the closest MRP1 homologue [94] and is located on chromosome 17q21.33. It is involved in resistance against the anti-cancer drugs etoposide, teniposide and at higher concentrations also to methotrexate [95]. MRP3 is located in basolateral plasma membranes of liver, adrenals, pancreas, kidney, gut and gallbladder. The physiological function of MRP3 is still unknown but its cellular localisation and the information on substrates implies a role for MRP3 in transport of organic anions from the liver into the blood, especially when secretion into bile is being blocked [96]. MRP3 protein and RNA was not detected in bronchial epithelium [4,96]. However, data in literature are conflicting and preliminary unpublished results indicate that Mrp3 is present in mouse lung. As already mentioned in the discussion on MRP1, primary epithelial cells (of bronchial and peripheral origin) and A549 cells stain positive for MRP3 (Figure 1) [85]. MRP3 mRNA was measured by quantitative RT-PCR in normal lung and lung tumours. MRP3 transcript levels were related to exposure to vincristine, etoposide and platinum drugs in lung tumours [97,98].

### MRP4 and MRP5

*MRP4 *(ABCC4) and *MRP5 *(ABCC5) genes map to 13q32.1 and 3q27.1 respectively. MRP4 protein is highly expressed in the kidney and prostate and MRP4 mRNA is present in the lung [55,99]. MRP5 mRNA is ubiquitously expressed, mainly in brain and skeletal muscle and also in the lung [6,55]. Primary epithelial cells (of bronchial and peripheral origin) and A549 cells stain positive for MRP4 and 5 (Figure 1) [85]. The substrate specificities of these two transporters differ from the other transporters of the ABCC family, i.e. they transport a variety of nucleoside analogues. The physiological role of both proteins is still unknown but they can serve as an efflux pump of the nucleosides cAMP and cGMP at low affinity, likely in a GSH independent manner [100-103]. Interestingly, AMP levels in lungs of asthma and COPD patients are elevated and therefore, MRP4 and MRP5 activity may be of clinical relevance in these diseases [104]. MRP4 can actively efflux prostaglandins E_1 _and E_2 _[105]. It was also suggested that MRP4 plays a role in the transport of conjugated steroids and bile acids [106], whereas MRP5 was reported to transport hyaluran out of cells [107]. In a recent study, a panel of 60 human cancer cell lines (the NCI-60) were screened with real-time RT-PCR for 48 human ABC transporters. Among these were several lung cancer cell lines. An association was found between MRP4 and MRP5 gene expression and resistance against platinum drugs in lung cancer [108-110].

### MRP6

MRP6 (ABCC6) maps to chromosome 16p13.12 and is mainly expressed in liver and kidney. MRP6 was neither detected in the lung nor in lung derived tumour cell lines SW1573 and GLC4 by immunohistochemistry [111]. However, MRP6 mRNA was moderately present in human lung extracts [6]. MRP6 is mutated in the hereditary connective tissue disorder pseudoxanthoma elasticum which affects skin, retina and blood vessels (for review, see [112]). Still, its physiological role in this disease and its substrate specificity is unclear. Pulmonary abnormalities are rare in pseudoxanthoma elasticum. However, in some patients calcification and elastic tissue damage in the lung have been described [113]. This may be due to an altered MRP6 function. Further studies are required to assess whether dysfunction of MRP6 also plays a role in development of other lung diseases such as emphysema. With *in situ *hybridisation and RNase protection assay in C57BL/6 mice, Mrp6 mRNA could be detected in tracheal and bronchial epithelium [114].

### MRP7, MRP8 and MRP9

To date, the functions of MRP7 (ABCC10, gene 6p21.1), MRP8 (ABCC11, gene 16q12.1) and MRP9 (ABCC12, gene 16q12.1) are largely unknown. Of these recently discovered ABC transporters, only MRP7 mRNA is highly expressed in total lung and trachea RNA extracts [6]. MRP7 function resembles P-gp function in the resistance against taxanes [115]. MRP8 resembles MRP4 function more than MRP5, and the physiological role of MRP8 may involve transport of conjugated steroids, cyclic nucleotides and bile acids [116].

### CFTR

#### CFTR localisation and function

*CFTR *(ABCC7) is located on chromosome 7q31.31. CFTR is the only member of the ABC superfamily which is not an active transporter. It functions as a chloride channel and in normal human airway tissue CFTR is highly expressed at the luminal side in serous cells of the submucosal glands. In addition, it is restricted to the apical membrane domain of well-differentiated epithelial cells such as ciliated cells, and probably also non-ciliated Clara cells and alveolar type I cells (Figure 1) [117-120]. CFTR is also expressed in normal nasal respiratory mucosa [13].

#### CFTR in cystic fibrosis

Mutations in the *CFTR *gene can cause cystic fibrosis [7] and are associated with abnormal Cl^- ^and Na^+ ^ion transport in several tissues including the lungs, pancreas, gastrointestinal tract, liver, sweat glands and male reproductive organs. Although the normal expression of CFTR in the lung is lower compared to tissues such as the intestine and pancreas, its function in the lung is of major importance. The most frequent mutation in this gene is the delta F508 mutation which leads to cystic fibrosis. Defective CFTR function can cause viscous secretions in the lungs which leads to chronic inflammation with acute exacerbations by impaired mucociliary clearance. There are major risks for colonisation with *Pseudonomas aeruginosa *which leads to pneumonia and respiratory insufficiency [121]. Besides abnormal CFTR localisation and expression in cystic fibrosis, also in non-cystic fibrosis airway tissue CFTR can be abnormally expressed in remodelled or dedifferentiated epithelium [122,123]. whereas in delta F508 CFTR epithelial cells there may be a normally processed CFTR [120,124]. The regulation and transport function of CFTR are dependent of the state of differentiation and polarisation of epithelial cell cultures [125,126]. Dedifferentiation with hyperplasia or metaplasia was associated with an intracellular localisation or absence of CFTR protein [122]. Hurbain *et al*. [127] analysed MRP1-5 transcript levels in nasal respiratory cells from cystic fibrosis patients with homozygous delta F508 mutation. Surprisingly, low MRP1 levels were associated with more severe disease and in addition, MRP1 levels were related to cAMP-independent chloride transport suggesting that MRP1 regulates another chloride channel in the apical membrane. CFTR function is cAMP regulated [121], and therefore, MRP4 or MRP5 activity may play a role in regulation of CFTR, since these proteins transport cAMP. Another study showed that CFTR function is blocked by two MRP substrates (taurolithocholate-3-sulphate and beta-estradiol) [128]. This implies that these two substances are also substrates for CFTR, or that CFTR and MRP proteins possess similar anion binding sites. Bebok *et al*. showed that nitric oxide (NO) and reactive oxygen nitrogen species (RONS) decrease wild-type CFTR protein levels in airway epithelial cell monolayers [129]. Natural sources of NO and RONS are activated in alveolar and interstitial macrophages [130,131], neutrophils [132], alveolar type II cells [133,134]. and airway epithelial cells [135]. In this view, CFTR function may be important in pulmonary infections and in the effect of oxidative stress generated by e.g. cigarette smoke.

#### CFTR in animal models

*Cftr *knock-out mice show reduced viability in contrast to most other ABC transporter knock-outs which are viable and fertile. Trezise *et al*. developed *Cftr *knock-out mice that have a severe cystic fibrosis phenotype accompanied by a lack of Cftr-related chloride conductance in e.g. tracheal epithelium [136,137]. They found that the P-gp mRNA level was four-fold increased in intestines of neonatal and 3- to 4-week-old *Cftr *knock-out mice compared to littermates of the same age. However, in 10 weeks-old mice P-gp levels were three-fold decreased. Apparently, a reduction or loss of Cftr function influences P-gp expression.

#### CFTR in vitro

The Calu-3 cell line, which has properties from serous cells of the pulmonary submucosal glands, is often used to study function and expression of CFTR [129,138]. It was demonstrated in Calu-3 cells that CFTR mRNA expression is downregulated after ouabain incubation whereas P-gp expression was upregulated [139]. The bronchial epithelial cell line 16HBE14o^- ^is also invaluable in CFTR research [129,140]. In wild-type and mutant CFTR expressing Sf9 insect cells, it was demonstrated that besides chloride transport, another function of CFTR is transport of GSH in a nucleotide-dependent manner [141]. This observation suggests that CFTR plays a role in the control of oxidative stress. Indeed, several studies in patients, mice and cell lines have shown that GSH levels are lower in case of defective CFTR function.

### BCRP

#### BCRP localisation and function

*BCRP *(ABCG2) is the breast cancer resistance protein (BCRP), located on chromosome 4q22. BCRP is a half-transporter that probably acts as a homo- or heterodimer [142] and is involved in resistance against toxins and several chemotherapeutic agents (e.g. mitoxantrone and topoisomerase 1 inhibitors) [143,144]. Protein levels of BCRP in the lung are lower than P-gp and MRP1 but distinct in the epithelial cell layer and in seromucinous glands (Figure 1) [4]. BCRP was absent in alveolar macrophages, suggesting that BCRP does not play a major role in innate inflammatory responses in the lung. Small endothelial capillaries also stain positive for BCRP, thus BCRP may protect the lungs against noxious compounds that enter systemically.

#### BCRP in tumours

In a study of untreated solid lung tumours, i.e. squamous cell carcinoma (n = 5), adenocarcinoma (n = 2) and SCLC (n = 3), most cases expressed moderate or strong BCRP [145]. In addition, higher BCRP expression in blood vessels was observed than in vessels of the surrounding tumour, indicating that BCRP may play a role in tumour angiogenesis. In 72 cases of advanced NSCLC, BCRP expression, but not P-gp, MRP1, MRP2 and MRP3, predicted poor clinical outcome [146].

#### BCRP in animal models

*Bcrp *knock-out mice do not display any abnormalities compared to wild-type mice under normal conditions, except for the colour of their bile which is red instead of yellow on certain diets [147]. However, these mice appear to be extremely sensitive to the phototoxin pheophorbide A, showing that BCRP is important to protect against toxic food components. Abnormalities of lungs in *Bcrp (-/-) *mice have not been reported thus far. Recently, Bcrp1 (Bcrp in the mouse is also called Bcrp1) localisation in the lung of 4- to 8-week old C57Bl/6J mice was studied to identify lung stem cells. These cells possess high activity of efflux of Hoechst dye that is transported by Bcrp1 [148]. In peripheral blood and bone marrow, cells with side population activity represent BCRP positive (haematopoietic) stem cells and this might also be the case for side population cells in the lungs. Indeed, *Bcrp1 (-/-) *mice did not display side population cells as shown by Hoechst staining of lung digests. In paraffin-embedded tissue sections, Bcrp1 was restricted to smooth muscle cells (of both arteries and airways) and a subpopulation of unidentified round cells in the alveolar space but not detectable in endothelial or epithelial cells. BAL cells of mice were Bcrp1 positive, but did not display side population (SP) activity [148]. Clara cells, a nonciliated bronchiolar epithelial cell type, were also phenotypically described to possess stem cell characteristics [149]. Further investigation is required to identify the stem cell pool in the lung in relation to expression of ABC transporters. Repairing and replacing lost lung tissue is a research area of promising therapeutic possibilities [150].

### ABCA1

The *ABCA1 *gene is mapped to chromosome 9q31.1. It is the causative gene in the development of Tangier disease, a disorder of cholesterol transport [8]. The ABCA1 protein controls transport of cholesterol and phospholipids to apolipoprotein 1 (apoA-1) in alveolar type II cells (Figure 1). Using the siRNA technique for ABCA1, it was shown that ABCA1 is involved in basolateral transport of surfactant that is activated by oxysterol [151]. *Abca1 *knock-out mice show increased concentrations of cholesterol precursors in lung, plasma, intestine and faeces [152]. It was demonstrated that there were major morphologic abnormalities in lungs of these mice, increasing with age. In 30% of 18 months old mice, lung parenchyma was affected. Lesions were characterised by foamy type II pneumocytes with aberrant lamellar bodies, intraalveolar macrophages and cholesterol clefts [153]. In addition, ABCA7, a close homologue of ABCA1 was also found to be highly expressed in mouse lung tissue by Western blot analysis [154].

### ABCA3

The *ABCA3 *gene is located on chromosome 16p13.3. The function of this ABC transporter has not been studied in detail but ABCA3 protein is present in lamellar bodies in human lung alveolar type II cells (Figure 1). Langmann *et al*. performed quantitative RT-PCR in 20 human tissues and found ABCA3 to be expressed restrictively in the lung [6]. In a recent study in cell lines, an association was found between lung cancer and ABCA3 (and also ABCA2) gene expression by means of quantitative RT-PCR [108].

The high expression of ABCA3 in alveolar type II cells suggests that ABCA3 may play a role in surfactant regulation [155]. Surfactant is important to lower the surface tension in the air-liquid interphase in alveoli. Indeed, in patients with surfactant deficiency and with severe neonatal lung disease it was demonstrated that the *ABCA3 *gene was frequently mutated [9]. *ABCA3 *mutations were found in 16 out of 21 patients, but polymorphisms (SNPs) were not found. One mutation was not fatal but was associated with a chronic lung disorder in a 6-year old patient. Other mutations turned out to be fatal and these patients died shortly after birth. Electron micrographs of tissue of patients with surfactant deficiency showed abnormal dense and small lamellar bodies. It was suggested that ABCA3 plays a role in phospholipid metabolism, since it closely resembles ABCA1 and ABCA4 that are known to transport phospholipids.

## Conclusion

Little is known about the function of ABC transporters that are expressed in the lung although their overall expression is very high compared to many other organs [6]. This review shows that ABC transporters in the lung are not only relevant for relatively rare diseases such as cystic fibrosis, Tangier disease and surfactant deficiency. Preliminary data indicate that MRP1 expression is lower in COPD patients than in healthy controls. Mutations and polymorphisms in ABC transporters may have important clinical consequences for development of lung diseases. However, overlap in substrate specificities may be compensatory in cases of malfunction of one (or more) transporter(s). Given the complexity of lung architecture, research on detailed cellular processes is difficult but challenging. Several ABC transporter deficient animal models have been developed that are of great value to study the role of these proteins. To date, exposure to cigarette smoke has never been tested in ABC transporter deficient animal models and would potentially give interesting information about the role of ABC transporters in protection against inhaled toxic substances such as present in tobacco smoke. Cell line models have been used to study transport processes and pulmonary drug metabolism. The delivery of pulmonary drugs to the site of action is probably highly dependent on the presence and activity of many ABC transporters in several cell types in the lung. The first barrier after inhalation is the pulmonary epithelium and transporters in the pulmonary endothelium may be critical for the delivery of intravenously or orally administered drugs. Insight in the function of ABC transporters in the lung may open new ways to facilitate treatment of lung diseases.

## Abbreviations

ABC = ATP-binding cassette

AMP = adenosine monophosphate

BALF = bronchoalveolar lavage fluid

B [a]P = benzo [a]pyrene

BCRP = breast cancer resistance protein

cAMP = cyclic adenosine monophosphate

CFTR = cystic fibrosis transmembrane conductance regulator

cGMP = cyclic guanosine monophosphate

cMOAT = canalicular multispecific organic anion transporter

CNS = central nervous sytem

COPD = chronic obstructive pulmonary disease

EHBR = Eisai hyperbilirubinemic rat

γ-GCS = gamma-glutamylcysteine synthetase

GSH = glutathione, reduced form

GSSG = glutathione disulfide; oxidized glutathione

GST = glutathione S-transferase

HPLC = high liquid performance chromatography

LTC_4 _= leuktotriene C_4_

LTB_4 _= leukotriene B_4_

MDR = multidrug resistance

MRP = multidrug resistance-associated protein

NSCLC = non-small cell lung cancer

NNAL = nitrosamine 4-(methylnitrosamino)-1-(3-pyridyl)-1-butanol

NNK = nitrosamine 4-(methylnitrosamino)-1-(3-pyridyl)-1-butanone

NO = nitric oxide

NRF2 = nuclear factor-E2 p45-related factor

P-gp = P-glycoprotein

R6G = rhodamine 6G

RONS = reactive oxygen nitrogen species

RT-PCR = reverse transcriptase-polymerase chain reaction

SCLC = small cell lung cancer

SNP = single nucleotide polymorphism

TKO = triple knock-out mice

## Competing interests

The author(s) declare that they have no competing interests.

## Authors' contributions

MD mainly drafted the manuscript, EV, WT, RS, HT and DP helped to draft the manuscript. All authors read and approved the final manuscript.

## References

[B1] Crapo JD, Barry BE, Gehr P, Bachofen M, Weibel ER (1982). Cell number and cell characteristics of the normal human lung. Am Rev Respir Dis.

[B2] Dean M, Hamon Y, Chimini G (2001). The human ATP-binding cassette (ABC) transporter superfamily. J Lipid Res.

[B3] Muller M Human ABC-transporters. http://nutrigene.4t.com/humanabc.htm.

[B4] Scheffer GL, Pijnenborg AC, Smit EF, Muller M, Postma DS, Timens W, van der Valk P, de Vries EG, Scheper RJ (2002). Multidrug resistance related molecules in human and murine lung. J Clin Pathol.

[B5] Hamilton KO, Yazdanian MA, Audus KL (2002). Contribution of efflux pump activity to the delivery of pulmonary therapeutics. Curr Drug Metab.

[B6] Langmann T, Mauerer R, Zahn A, Moehle C, Probst M, Stremmel W, Schmitz G (2003). Real-time reverse transcription-PCR expression profiling of the complete human ATP-binding cassette transporter superfamily in various tissues. Clin Chem.

[B7] Riordan JR, Rommens JM, Kerem B, Alon N, Rozmahel R, Grzelczak Z, Zielenski J, Lok S, Plavsic N, Chou JL (1989). Identification of the cystic fibrosis gene: cloning and characterization of complementary DNA. Science.

[B8] Brooks-Wilson A, Marcil M, Clee SM, Zhang LH, Roomp K, van Dam M, Yu L, Brewer C, Collins JA, Molhuizen HO, Loubser O, Ouelette BF, Fichter K, Ashbourne-Excoffon KJ, Sensen CW, Scherer S, Mott S, Denis M, Martindale D, Frohlich J, Morgan K, Koop B, Pimstone S, Kastelein JJ, Hayden MR (1999). Mutations in ABC1 in Tangier disease and familial high-density lipoprotein deficiency. Nat Genet.

[B9] Shulenin S, Nogee LM, Annilo T, Wert SE, Whitsett JA, Dean M (2004). ABCA3 gene mutations in newborns with fatal surfactant deficiency. N Engl J Med.

[B10] Van der Deen M, Marks H, Muller M, Pijnenborg AC, Postma DS, Scheffer G, Scheper RJ, Smit EF, Timens W, de Vries EG (2003). Diminished expression of Multidrug Resistance-associated Protein 1 (MRP1) in bronchial epithelium of COPD patients [abstract]. Am J Respir Crit Care Med.

[B11] Gottesman MM, Fojo T, Bates SE (2002). Multidrug resistance in cancer: role of ATP-dependent transporters. Nat Rev Cancer.

[B12] Lechapt-Zalcman E, Hurbain I, Lacave R, Commo F, Urban T, Antoine M, Milleron B, Bernaudin JF (1997). MDR1-Pgp 170 expression in human bronchus. Eur Respir J.

[B13] Wioland MA, Fleury-Feith J, Corlieu P, Commo F, Monceaux G, Lacau-St-Guily J, Bernaudin JF (2000). CFTR, MDR1, and MRP1 immunolocalization in normal human nasal respiratory mucosa. J Histochem Cytochem.

[B14] Campbell L, Abulrob AN, Kandalaft LE, Plummer S, Hollins AJ, Gibbs A, Gumbleton M (2003). Constitutive expression of p-glycoprotein in normal lung alveolar epithelium and functionality in primary alveolar epithelial cultures. J Pharmacol Exp Ther.

[B15] Cordon-Cardo C, O'Brien JP, Boccia J, Casals D, Bertino JR, Melamed MR (1990). Expression of the multidrug resistance gene product (P-glycoprotein) in human normal and tumor tissues. J Histochem Cytochem.

[B16] Van der Valk P, van Kalken CK, Ketelaars H, Broxterman HJ, Scheffer G, Kuiper CM, Tsuruo T, Lankelma J, Meijer CJ, Pinedo HM (1990). Distribution of multi-drug resistance-associated P-glycoprotein in normal and neoplastic human tissues. Analysis with 3 monoclonal antibodies recognizing different epitopes of the P-glycoprotein molecule. Ann Oncol.

[B17] Puddu P, Fais S, Luciani F, Gherardi G, Dupuis ML, Romagnoli G, Ramoni C, Cianfriglia M, Gessani S (1999). Interferon-gamma up-regulates expression and activity of P-glycoprotein in human peripheral blood monocyte-derived macrophages. Lab Invest.

[B18] Valverde MA, Diaz M, Sepulveda FV, Gill DR, Hyde SC, Higgins CF (1992). Volume-regulated chloride channels associated with the human multidrug-resistance P-glycoprotein. Nature.

[B19] Gill DR, Hyde SC, Higgins CF, Valverde MA, Mintenig GM, Sepulveda FV (1992). Separation of drug transport and chloride channel functions of the human multidrug resistance P-glycoprotein. Cell.

[B20] Higgins CF (1995). P-glycoprotein and cell volume-activated chloride channels. J Bioenerg Biomembr.

[B21] Kunzelmann K, Slotki IN, Klein P, Koslowsky T, Ausiello DA, Greger R, Cabantchik ZI (1994). Effects of P-glycoprotein expression on cyclic AMP and volume-activated ion fluxes and conductances in HT-29 colon adenocarcinoma cells. J Cell Physiol.

[B22] De Greef C, Sehrer J, Viana F, van Acker K, Eggermont J, Mertens L, Raeymaekers L, Droogmans G, Nilius B (1995). Volume-activated chloride currents are not correlated with P-glycoprotein expression. Biochem J.

[B23] Waters CM, Krejcie TC, Avram MJ (2000). Facilitated uptake of fentanyl, but not alfentanil, by human pulmonary endothelial cells. Anesthesiology.

[B24] Wood L, Palmer M, Hewitt J, Urtasun R, Bruera E, Rapp E, Thaell JF (1998). Results of a phase III, double-blind, placebo-controlled trial of megestrol acetate modulation of P-glycoprotein-mediated drug resistance in the first-line management of small-cell lung carcinoma. Br J Cancer.

[B25] Beer TW, Rowlands DC, Crocker J (1996). Detection of the multidrug resistance marker P-glycoprotein by immunohistochemistry in malignant lung tumours. Thorax.

[B26] Volm M, Mattern J, Samsel B (1992). Relationship of inherent resistance to doxorubicin, proliferative activity and expression of P-glycoprotein 170, and glutathione S-transferase-pi in human lung tumors. Cancer.

[B27] Volm M, Mattern J, Samsel B (1991). Overexpression of P-glycoprotein and glutathione S-transferase-pi in resistant non-small cell lung carcinomas of smokers. Br J Cancer.

[B28] Hoffmeyer S, Burk O, von Richter O, Arnold HP, Brockmoller J, Johne A, Cascorbi I, Gerloff T, Roots I, Eichelbaum M, Brinkmann U (2000). Functional polymorphisms of the human multidrug-resistance gene: multiple sequence variations and correlation of one allele with P-glycoprotein expression and activity in vivo. Proc Natl Acad Sci U S A.

[B29] Eichelbaum M, Fromm MF, Schwab M (2004). Clinical aspects of the MDR1 (ABCB1) gene polymorphism. Ther Drug Monit.

[B30] Johannesson M, Nordqvist AC, Bogdanovic N, Hjelte L, Schalling M (1997). Polymorphic expression of multidrug resistance mRNA in lung parenchyma of nonpregnant and pregnant rats: a comparison to cystic fibrosis mRNA expression. Biochem Biophys Res Commun.

[B31] Sinues B, Fanlo A, Bernal ML, Mayayo E, Bello S, Rubio E, Isla D (2003). MDR-1 C3435T genetic polymorphism and tobacco-related lung cancer. Oncology.

[B32] Isla D, Sarries C, Rosell R, Alonso G, Domine M, Taron M, Lopez-Vivanco G, Camps C, Botia M, Nunez L, Sanchez-Ronco M, Sanchez JJ, Lopez-Brea M, Barneto I, Paredes A, Medina B, Artal A, Lianes P (2004). Single nucleotide polymorphisms and outcome in docetaxel-cisplatin-treated advanced non-small-cell lung cancer. Ann Oncol.

[B33] Zheng H, Zeevi A, Schuetz E, Lamba J, McCurry K, Griffith BP, Webber S, Ristich J, Dauber J, Iacono A, Grgurich W, Zaldonis D, McDade K, Zhang J, Burckart GJ (2004). Tacrolimus dosing in adult lung transplant patients is related to cytochrome P4503A5 gene polymorphism. J Clin Pharmacol.

[B34] Brady JM, Cherrington NJ, Hartley DP, Buist SC, Li N, Klaassen CD (2002). Tissue distribution and chemical induction of multiple drug resistance genes in rats. Drug Metab Dispos.

[B35] Seree E, Villard PH, Hever A, Guigal N, Puyoou F, Charvet B, Point-Scomma H, Lechevalier E, Lacarelle B, Barra Y (1998). Modulation of MDR1 and CYP3A expression by dexamethasone: evidence for an inverse regulation in adrenals. Biochem Biophys Res Commun.

[B36] Hendrikse NH, de Vries EG, Eriks-Fluks L, van der Graaf WT, Hospers GA, Willemsen AT, Vaalburg W, Franssen EJ (1999). A new in vivo method to study P-glycoprotein transport in tumors and the blood-brain barrier. Cancer Res.

[B37] Roerig DL, Audi SH, Ahlf SB (2004). Kinetic characterization of p-glycoprotein-mediated efflux of rhodamine 6g in the intact rabbit lung. Drug Metab Dispos.

[B38] Booth CL, Brouwer KR, Brouwer KL (1998). Effect of multidrug resistance modulators on the hepatobiliary disposition of doxorubicin in the isolated perfused rat liver. Cancer Res.

[B39] Schinkel AH, Mayer U, Wagenaar E, Mol CA, van Deemter L, Smit JJ, van der Valk MA, Voordouw AC, Spits H, van Tellingen O, Zijlmans JM, Fibbe WE, Borst P (1997). Normal viability and altered pharmacokinetics in mice lacking mdr1-type (drug-transporting) P-glycoproteins. Proc Natl Acad Sci U S A.

[B40] Grauer MT, Uhr M (2004). P-glycoprotein reduces the ability of amitriptyline metabolites to cross the blood brain barrier in mice after a 10-day administration of amitriptyline. J Psychopharmacol.

[B41] Forbes II (2000). Human airway epithelial cell lines for in vitro drug transport and metabolism studies. Pharm Sci Technol Today.

[B42] Ehrhardt C, Kneuer C, Laue M, Schaefer UF, Kim KJ, Lehr CM (2003). 16HBE14o- human bronchial epithelial cell layers express P-glycoprotein, lung resistance-related protein, and caveolin-1. Pharm Res.

[B43] Hamilton KO, Yazdanian MA, Audus KL (2001). Modulation of P-glycoprotein activity in Calu-3 cells using steroids and beta-ligands. Int J Pharm.

[B44] Hamilton KO, Topp E, Makagiansar I, Siahaan T, Yazdanian M, Audus KL (2001). Multidrug resistance-associated protein-1 functional activity in Calu-3 cells. J Pharmacol Exp Ther.

[B45] Lehmann T, Kohler C, Weidauer E, Taege C, Foth H (2001). Expression of MRP1 and related transporters in human lung cells in culture. Toxicology.

[B46] Cole SP, Bhardwaj G, Gerlach JH, Mackie JE, Grant CE, Almquist KC, Stewart AJ, Kurz EU, Duncan AM, Deeley RG (1992). Overexpression of a transporter gene in a multidrug-resistant human lung cancer cell line. Science.

[B47] Muller M, Meijer C, Zaman GJ, Borst P, Scheper RJ, Mulder NH, de Vries EG, Jansen PL (1994). Overexpression of the gene encoding the multidrug resistance-associated protein results in increased ATP-dependent glutathione S-conjugate transport. Proc Natl Acad Sci U S A.

[B48] Hipfner DR, Deeley RG, Cole SP (1999). Structural, mechanistic and clinical aspects of MRP1. Biochim Biophys Acta.

[B49] Loe DW, Deeley RG, Cole SP (1998). Characterization of vincristine transport by the M(r) 190,000 multidrug resistance protein (MRP): evidence for cotransport with reduced glutathione. Cancer Res.

[B50] Renes J, de Vries EG, Nienhuis EF, Jansen PL, Muller M (1999). ATP- and glutathione-dependent transport of chemotherapeutic drugs by the multidrug resistance protein MRP1. Br J Pharmacol.

[B51] Hooijberg JH, Broxterman HJ, Kool M, Assaraf YG, Peters GJ, Noordhuis P, Scheper RJ, Borst P, Pinedo HM, Jansen G (1999). Antifolate resistance mediated by the multidrug resistance proteins MRP1 and MRP2. Cancer Res.

[B52] Leier I, Jedlitschky G, Buchholz U, Keppler D (1994). Characterization of the ATP-dependent leukotriene C4 export carrier in mastocytoma cells. Eur J Biochem.

[B53] Brechot JM, Hurbain I, Fajac A, Daty N, Bernaudin JF (1998). Different pattern of MRP localization in ciliated and basal cells from human bronchial epithelium. J Histochem Cytochem.

[B54] Flens MJ, Zaman GJ, van der Valk P, Izquierdo MA, Schroeijers AB, Scheffer GL, van der Groep P, de Haas M, Meijer CJ, Scheper RJ (1996). Tissue distribution of the multidrug resistance protein. Am J Pathol.

[B55] Kool M, de Haas M, Scheffer GL, Scheper RJ, van Eijk MJ, Juijn JA, Baas F, Borst P (1997). Analysis of expression of cMOAT (MRP2), MRP3, MRP4, and MRP5, homologues of the multidrug resistance-associated protein gene (MRP1), in human cancer cell lines. Cancer Res.

[B56] Wijnholds J, Scheffer GL, van der Valk M, van der Valk P, Beijnen JH, Scheper RJ, Borst P (1998). Multidrug resistance protein 1 protects the oropharyngeal mucosal layer and the testicular tubules against drug-induced damage. J Exp Med.

[B57] Thomas GA, Barrand MA, Stewart S, Rabbitts PH, Williams ED, Twentyman PR (1994). Expression of the multidrug resistance-associated protein (MRP) gene in human lung tumours and normal tissue as determined by in situ hybridisation. Eur J Cancer.

[B58] Doubre H, Cesari D, Mairovitz A, Benac C, Chantot-Bastaraud S, Dagnon K, Antoine M, Danel C, Bernaudin JF, Fleury-Feith J (2005). Multidrug resistance-associated protein (MRP1) is overexpressed in DNA aneuploid carcinomatous cells in non-small cell lung cancer (NSCLC). Int J Cancer.

[B59] Trompier D, Chang XB, Barattin R, du Moulinet d'Hardemare A, Di Pietro A, Baubichon-Cortay H (2004). Verapamil and its derivative trigger apoptosis through glutathione extrusion by multidrug resistance protein MRP1. Cancer Res.

[B60] Leslie EM, Ito K, Upadhyaya P, Hecht SS, Deeley RG, Cole SP (2001). Transport of the beta -O-glucuronide conjugate of the tobacco-specific carcinogen 4-(methylnitrosamino)-1-(3-pyridyl)-1-butanol (NNAL) by the multidrug resistance protein 1 (MRP1). Requirement for glutathione or a non-sulfur-containing analog. J Biol Chem.

[B61] Kerb R, Hoffmeyer S, Brinkmann U (2001). ABC drug transporters: hereditary polymorphisms and pharmacological impact in MDR1, MRP1 and MRP2. Pharmacogenomics.

[B62] Saito S, Iida A, Sekine A, Miura Y, Ogawa C, Kawauchi S, Higuchi S, Nakamura Y (2002). Identification of 779 genetic variations in eight genes encoding members of the ATP-binding cassette, subfamily C (ABCC/MRP/CFTR. J Hum Genet.

[B63] Oselin K, Mrozikiewicz PM, Gaikovitch E, Pahkla R, Roots I (2003). Frequency of MRP1 genetic polymorphisms and their functional significance in Caucasians: detection of a novel mutation G816A in the human MRP1 gene. Eur J Clin Pharmacol.

[B64] Ito S, Ieiri I, Tanabe M, Suzuki A, Higuchi S, Otsubo K (2001). Polymorphism of the ABC transporter genes, MDR1, MRP1 and MRP2/cMOAT, in healthy Japanese subjects. Pharmacogenetics.

[B65] Ito K, Olsen SL, Qiu W, Deeley RG, Cole SP (2001). Mutation of a single conserved tryptophan in multidrug resistance protein 1 (MRP1/ABCC1) results in loss of drug resistance and selective loss of organic anion transport. J Biol Chem.

[B66] Conrad S, Kauffmann HM, Ito K, Leslie EM, Deeley RG, Schrenk D, Cole SP (2002). A naturally occurring mutation in MRP1 results in a selective decrease in organic anion transport and in increased doxorubicin resistance. Pharmacogenetics.

[B67] Lorico A, Rappa G, Finch RA, Yang D, Flavell RA, Sartorelli AC (1997). Disruption of the murine MRP (multidrug resistance protein) gene leads to increased sensitivity to etoposide (VP-16) and increased levels of glutathione. Cancer Res.

[B68] Wijnholds J, Evers R, van Leusden MR, Mol CA, Zaman GJ, Mayer U, Beijnen JH, van der Valk M, Krimpenfort P, Borst P (1997). Increased sensitivity to anticancer drugs and decreased inflammatory response in mice lacking the multidrug resistance-associated protein. Nat Med.

[B69] Bain LJ, Feldman RA (2003). Altered expression of sulfotransferases, glucuronosyltransferases and mrp transporters in FVB/mrp1-/- mice. Xenobiotica.

[B70] Verbon A, Leemans JC, Weijer S, Florquin S, van der PT (2002). Mice lacking the multidrug resistance protein 1 have a transiently impaired immune response during tuberculosis. Clin Exp Immunol.

[B71] Schultz MJ, Wijnholds J, Peppelenbosch MP, Vervoordeldonk MJ, Speelman P, van Deventer SJ, Borst P, van der Poll T (2001). Mice lacking the multidrug resistance protein 1 are resistant to Streptococcus pneumoniae-induced pneumonia. J Immunol.

[B72] Crooks SW, Bayley DL, Hill SL, Stockley RA (2000). Bronchial inflammation in acute bacterial exacerbations of chronic bronchitis: the role of leukotriene B4. Eur Respir J.

[B73] Biernacki WA, Kharitonov SA, Barnes PJ (2003). Increased leukotriene B4 and 8-isoprostane in exhaled breath condensate of patients with exacerbations of COPD. Thorax.

[B74] Loe DW, Stewart RK, Massey TE, Deeley RG, Cole SP (1997). ATP-dependent transport of aflatoxin B1 and its glutathione conjugates by the product of the multidrug resistance protein (MRP) gene. Mol Pharmacol.

[B75] Lorico A, Nesland J, Emilsen E, Fodstad O, Rappa G (2002). Role of the multidrug resistance protein 1 gene in the carcinogenicity of aflatoxin B1: investigations using mrp1-null mice. Toxicology.

[B76] Hayashi A, Suzuki H, Itoh K, Yamamoto M, Sugiyama Y (2003). Transcription factor Nrf2 is required for the constitutive and inducible expression of multidrug resistance-associated protein 1 in mouse embryo fibroblasts. Biochem Biophys Res Commun.

[B77] Rangasamy T, Cho CY, Thimmulappa RK, Zhen L, Srisuma SS, Kensler TW, Yamamoto M, Petrache I, Tuder RM, Biswal S (2004). Genetic ablation of Nrf2 enhances susceptibility to cigarette smoke-induced emphysema in mice. J Clin Invest.

[B78] Yamamoto T, Yoh K, Kobayashi A, Ishii Y, Kure S, Koyama A, Sakamoto T, Sekizawa K, Motohashi H, Yamamoto M (2004). Identification of polymorphisms in the promoter region of the human NRF2 gene. Biochem Biophys Res Commun.

[B79] Wijnholds J, deLange EC, Scheffer GL, van den Berg DJ, Mol CA, van der Valk M, Schinkel AH, Scheper RJ, Breimer DD, Borst P (2000). Multidrug resistance protein 1 protects the choroid plexus epithelium and contributes to the blood-cerebrospinal fluid barrier. J Clin Invest.

[B80] Johnson DR, Finch RA, Lin ZP, Zeiss CJ, Sartorelli AC (2001). The pharmacological phenotype of combined multidrug-resistance mdr1a/1b- and mrp1-deficient mice. Cancer Res.

[B81] Muramatsu T, Johnson DR, Finch RA, Johnson LK, Leffert JJ, Lin ZP, Pizzorno G, Sartorelli AC (2004). Age-related differences in vincristine toxicity and biodistribution in wild-type and transporter-deficient mice. Oncol Res.

[B82] Bandi N, Kompella UB (2002). Budesonide reduces multidrug resistance-associated protein 1 expression in an airway epithelial cell line (Calu-1). Eur J Pharmacol.

[B83] Van der Deen M, Timmer-Bosscha H, Timens W, Postma DS, de Vries EG (2004). Effect of cigarette smoke extract on MRP1 function in bronchial epithelial cells [abstract]. Proc Amer Assoc Cancer Res.

[B84] Nagayoshi K, Nemoto T, Yokoyama S, Yamashita F, Hashida M (2004). Effect of polycyclic aromatic hydrocarbons on generation and efflux of glutathione conjugates in primary cultured alveolar epithelial cells. Drug Metab Pharmacokinet.

[B85] Torky AR, Stehfest E, Viehweger K, Taege C, Foth H (2005). Immuno-histochemical detection of MRPs in human lung cells in culture. Toxicology.

[B86] Buchler M, Konig J, Brom M, Kartenbeck J, Spring H, Horie T, Keppler D (1996). cDNA cloning of the hepatocyte canalicular isoform of the multidrug resistance protein, cMrp, reveals a novel conjugate export pump deficient in hyperbilirubinemic mutant rats. J Biol Chem.

[B87] Cui Y, Konig J, Buchholz JK, Spring H, Leier I, Keppler D (1999). Drug resistance and ATP-dependent conjugate transport mediated by the apical multidrug resistance protein, MRP2, permanently expressed in human and canine cells. Mol Pharmacol.

[B88] Leier I, Jedlitschky G, Buchholz U, Cole SP, Deeley RG, Keppler D (1994). The MRP gene encodes an ATP-dependent export pump for leukotriene C4 and structurally related conjugates. J Biol Chem.

[B89] Kamisako T, Leier I, Cui Y, Konig J, Buchholz U, Hummel-Eisenbeiss J, Keppler D (1999). Transport of monoglucuronosyl and bisglucuronosyl bilirubin by recombinant human and rat multidrug resistance protein 2. Hepatology.

[B90] Holland IB, Cole SP, Kuchler K, Higgins CF (2003). ABC proteins, from bacteria to men.

[B91] Paulusma CC, Bosma PJ, Zaman GJ, Bakker CT, Otter M, Scheffer GL, Scheper RJ, Borst P, Oude Elferink RP (1996). Congenital jaundice in rats with a mutation in a multidrug resistance-associated protein gene. Science.

[B92] Hosokawa S, Tagaya O, Mikami T, Nozaki Y, Kawaguchi A, Yamatsu K, Shamoto M (1992). A new rat mutant with chronic conjugated hyperbilirubinemia and renal glomerular lesions. Lab Anim Sci.

[B93] Kuroda M, Kobayashi Y, Tanaka Y, Itani T, Mifuji R, Araki J, Kaito M, Adachi Y (2004). Increased hepatic and renal expressions of multidrug resistance-associated protein 3 in Eisai hyperbilirubinuria rats. J Gastroenterol Hepatol.

[B94] Kiuchi Y, Suzuki H, Hirohashi T, Tyson CA, Sugiyama Y (1998). cDNA cloning and inducible expression of human multidrug resistance associated protein 3 (MRP3). FEBS Lett.

[B95] Kool M, van der Linden M, de Haas M, Scheffer GL, de Vree JM, Smith AJ, Jansen G, Peters GJ, Ponne N, Scheper RJ, Elferink RP, Baas F, Borst P (1999). MRP3, an organic anion transporter able to transport anti-cancer drugs. Proc Natl Acad Sci U S A.

[B96] Scheffer GL, Kool M, de Haas M, de Vree JM, Pijnenborg AC, Bosman DK, Elferink RP, van der Valk P, Borst P, Scheper RJ (2002). Tissue distribution and induction of human multidrug resistant protein 3. Lab Invest.

[B97] Oguri T, Isobe T, Fujitaka K, Ishikawa N, Kohno N (2001). Association between expression of the MRP3 gene and exposure to platinum drugs in lung cancer. Int J Cancer.

[B98] Young LC, Campling BG, Voskoglou-Nomikos T, Cole SP, Deeley RG, Gerlach JH (1999). Expression of multidrug resistance protein-related genes in lung cancer: correlation with drug response. Clin Cancer Res.

[B99] Borst P, Evers R, Kool M, Wijnholds J (2000). A family of drug transporters: the multidrug resistance-associated proteins. J Natl Cancer Inst.

[B100] Wielinga PR, van der Heijden I, Reid G, Beijnen JH, Wijnholds J, Borst P (2003). Characterization of the MRP4- and MRP5-mediated transport of cyclic nucleotides from intact cells. J Biol Chem.

[B101] Jedlitschky G, Burchell B, Keppler D (2000). The multidrug resistance protein 5 functions as an ATP-dependent export pump for cyclic nucleotides. J Biol Chem.

[B102] Chen ZS, Lee K, Kruh GD (2001). Transport of cyclic nucleotides and estradiol 17-beta-D-glucuronide by multidrug resistance protein 4. Resistance to 6-mercaptopurine and 6-thioguanine. J Biol Chem.

[B103] Van Aubel RA, Smeets PH, Peters JG, Bindels RJ, Russel FG (2002). The MRP4/ABCC4 gene encodes a novel apical organic anion transporter in human kidney proximal tubules: putative efflux pump for urinary cAMP and cGMP. J Am Soc Nephrol.

[B104] Van den Berge M, Polosa R, Kerstjens HA, Postma DS (2004). The role of endogenous and exogenous AMP in asthma and chronic obstructive pulmonary disease. J Allergy Clin Immunol.

[B105] Reid G, Wielinga P, Zelcer N, van der Heijden I, Kuil A, de Haas M, Wijnholds J, Borst P (2003). The human multidrug resistance protein MRP4 functions as a prostaglandin efflux transporter and is inhibited by nonsteroidal antiinflammatory drugs. Proc Natl Acad Sci USA.

[B106] Zelcer N, Reid G, Wielinga P, Kuil A, van der Heijden I, Schuetz JD, Borst P (2003). Steroid and bile acid conjugates are substrates of human multidrug-resistance protein (MRP) 4 (ATP-binding cassette C4). Biochem J.

[B107] Prehm P, Schumacher U (2004). Inhibition of hyaluronan export from human fibroblasts by inhibitors of multidrug resistance transporters. Biochem Pharmacol.

[B108] Szakacs G, Annereau JP, Lababidi S, Shankavaram U, Arciello A, Bussey KJ, Reinhold W, Guo Y, Kruh GD, Reimers M, Weinstein JN, Gottesman MM (2004). Predicting drug sensitivity and resistance; Profiling ABC transporter genes in cancer cells. Cancer Cell.

[B109] Savaraj N, Wu C, Wangpaichitr M, Kuo MT, Lampidis T, Robles C, Furst AJ, Feun L (2003). Overexpression of mutated MRP4 in cisplatin resistant small cell lung cancer cell line: collateral sensitivity to azidothymidine. Int J Oncol.

[B110] Suzuki T, Nishio K, Tanabe S (2001). The MRP family and anticancer drug metabolism. Curr Drug Metab.

[B111] Scheffer GL, Hu X, Pijnenborg AC, Wijnholds J, Bergen AA, Scheper RJ (2002). MRP6 (ABCC6) detection in normal human tissues and tumors. Lab Invest.

[B112] Hu X, Plomp AS, van Soest S, Wijnholds J, de Jong PT, Bergen AA (2003). Pseudoxanthoma elasticum: a clinical, histopathological, and molecular update. Surv Ophthalmol.

[B113] Jackson A, Loh CL (1980). Pulmonary calcification and elastic tissue damage in pseudoxanthoma elasticum. Histopathology.

[B114] Beck K, Hayashi K, Nishiguchi B, Le Saux O, Hayashi M, Boyd CD (2003). The distribution of Abcc6 in normal mouse tissues suggests multiple functions for this ABC transporter. J Histochem Cytochem.

[B115] Hopper-Borge E, Chen ZS, Shchaveleva I, Belinsky MG, Kruh GD (2004). Analysis of the drug resistance profile of multidrug resistance protein 7 (ABCC10): resistance to docetaxel. Cancer Res.

[B116] Chen ZS, Guo Y, Belinsky MG, Kotova E, Kruh GD (2005). Transport of bile acids, sulfated steroids, estradiol 17-beta-D-glucuronide, and leukotriene C4 by human multidrug resistance protein 8 (ABCC11). Mol Pharmacol.

[B117] Denning GM, Ostedgaard LS, Welsh MJ (1992). Abnormal localization of cystic fibrosis transmembrane conductance regulator in primary cultures of cystic fibrosis airway epithelia. J Cell Biol.

[B118] Engelhardt JF, Zepeda M, Cohn JA, Yankaskas JR, Wilson JM (1994). Expression of the cystic fibrosis gene in adult human lung. J Clin Invest.

[B119] Puchelle E, Gaillard D, Ploton D, Hinnrasky J, Fuchey C, Boutterin MC, Jacquot J, Dreyer D, Pavirani A, Dalemans W (1992). Differential localization of the cystic fibrosis transmembrane conductance regulator in normal and cystic fibrosis airway epithelium. Am J Respir Cell Mol Biol.

[B120] Kalin N, Claass A, Sommer M, Puchelle E, Tummler B (1999). DeltaF508 CFTR protein expression in tissues from patients with cystic fibrosis. J Clin Invest.

[B121] Pilewski JM, Frizzell RA (1999). Role of CFTR in airway disease. Physiol Rev.

[B122] Brezillon S, Dupuit F, Hinnrasky J, Marchand V, Kalin N, Tummler B, Puchelle E (1995). Decreased expression of the CFTR protein in remodeled human nasal epithelium from non-cystic fibrosis patients. Lab Invest.

[B123] Brezillon S, Hamm H, Heilmann M, Schafers HJ, Hinnrasky J, Wagner TO, Puchelle E, Tummler B (1997). Decreased expression of the cystic fibrosis transmembrane conductance regulator protein in remodeled airway epithelium from lung transplanted patients. Hum Pathol.

[B124] Penque D, Mendes F, Beck S, Farinha C, Pacheco P, Nogueira P, Lavinha J, Malho R, Amaral MD (2000). Cystic fibrosis F508del patients have apically localized CFTR in a reduced number of airway cells. Lab Invest.

[B125] Morris AP, Cunningham SA, Benos DJ, Frizzell RA (1992). Cellular differentiation is required for cAMP but not Ca(2+)-dependent Cl- secretion in colonic epithelial cells expressing high levels of cystic fibrosis transmembrane conductance regulator. J Biol Chem.

[B126] Hollande E, Fanjul M, Chemin-Thomas C, Devaux C, Demolombe S, Van Rietschoten J, Guy-Crotte O, Figarella C (1998). Targeting of CFTR protein is linked to the polarization of human pancreatic duct cells in culture. Eur J Cell Biol.

[B127] Hurbain I, Sermet-Gaudelus I, Vallee B, Feuillet MN, Lenoir G, Bernaudin JF, Edelman A, Fajac A (2003). Evaluation of MRP1-5 gene expression in cystic fibrosis patients homozygous for the delta F508 mutation. Pediatr Res.

[B128] Linsdell P, Hanrahan JW (1999). Substrates of multidrug resistance-associated proteins block the cystic fibrosis transmembrane conductance regulator chloride channel. Br J Pharmacol.

[B129] Bebok Z, Varga K, Hicks JK, Venglarik CJ, Kovacs T, Chen L, Hardiman KM, Collawn JF, Sorscher EJ, Matalon S (2002). Reactive oxygen nitrogen species decrease cystic fibrosis transmembrane conductance regulator expression and cAMP-mediated Cl- secretion in airway epithelia. J Biol Chem.

[B130] Hickman-Davis J, Gibbs-Erwin J, Lindsey JR, Matalon S (1999). Surfactant protein A mediates mycoplasmacidal activity of alveolar macrophages by production of peroxynitrite. Proc Natl Acad Sci U S A.

[B131] Hickman-Davis JM, O'Reilly P, Davis IC, Peti-Peterdi J, Davis G, Young KR, Devlin RB, Matalon S (2002). Killing of Klebsiella pneumoniae by human alveolar macrophages. Am J Physiol Lung Cell Mol Physiol.

[B132] Eiserich JP, Hristova M, Cross CE, Jones AD, Freeman BA, Halliwell B, van der Vliet A (1998). Formation of nitric oxide-derived inflammatory oxidants by myeloperoxidase in neutrophils. Nature.

[B133] Punjabi CJ, Laskin JD, Pendino KJ, Goller NL, Durham SK, Laskin DL (1994). Production of nitric oxide by rat type II pneumocytes: increased expression of inducible nitric oxide synthase following inhalation of a pulmonary irritant. Am J Respir Cell Mol Biol.

[B134] Weinberger B, Heck DE, Laskin DL, Laskin JD (1999). Nitric oxide in the lung: therapeutic and cellular mechanisms of action. Pharmacol Ther.

[B135] Meng QH, Polak JM, Edgar AJ, Chacon MR, Evans TJ, Gruenert DC, Bishop AE (2000). Neutrophils enhance expression of inducible nitric oxide synthase in human normal but not cystic fibrosis bronchial epithelial cells. J Pathol.

[B136] Trezise AE, Ratcliff R, Hawkins TE, Evans MJ, Freeman TC, Romano PR, Higgins CF, Colledge WH (1997). Co-ordinate regulation of the cystic fibrosis and multidrug resistance genes in cystic fibrosis knockout mice. Hum Mol Genet.

[B137] Ratcliff R, Evans MJ, Cuthbert AW, MacVinish LJ, Foster D, Anderson JR, Colledge WH (1993). Production of a severe cystic fibrosis mutation in mice by gene targeting. Nat Genet.

[B138] Shen BQ, Finkbeiner WE, Wine JJ, Mrsny RJ, Widdicombe JH (1994). Calu-3: a human airway epithelial cell line that shows cAMP-dependent Cl- secretion. Am J Physiol.

[B139] Baudouin-Legros M, Brouillard F, Tondelier D, Hinzpeter A, Edelman A (2003). Effect of ouabain on CFTR gene expression in human Calu-3 cells. Am J Physiol Cell Physiol.

[B140] Cozens AL, Yezzi MJ, Kunzelmann K, Ohrui T, Chin L, Eng K, Finkbeiner WE, Widdicombe JH, Gruenert DC (1994). CFTR expression and chloride secretion in polarized immortal human bronchial epithelial cells. Am J Respir Cell Mol Biol.

[B141] Kogan I, Ramjeesingh M, Li C, Kidd JF, Wang Y, Leslie EM, Cole SP, Bear CE (2003). CFTR directly mediates nucleotide-regulated glutathione flux. EMBO J.

[B142] Croop JM, Tiller GE, Fletcher JA, Lux ML, Raab E, Goldenson D, Son D, Arciniegas S, Wu RL (1997). Isolation and characterization of a mammalian homolog of the Drosophila white gene. Gene.

[B143] Doyle LA, Yang W, Abruzzo LV, Krogmann T, Gao Y, Rishi AK, Ross DD (1998). A multidrug resistance transporter from human MCF-7 breast cancer cells. Proc Natl Acad Sci U S A.

[B144] Komatani H, Kotani H, Hara Y, Nakagawa R, Matsumoto M, Arakawa H, Nishimura S (7088). Identification of breast cancer resistant protein/mitoxantrone resistance/placenta-specific, ATP-binding cassette transporter as a transporter of NB-506 and J-10 topoisomerase I inhibitors with an indolocarbazole structure. Cancer Res.

[B145] Diestra JE, Scheffer GL, Catala II, Maliepaard M, Schellens JH, Scheper RJ, Germa-Lluch JR, Izquierdo MA (2002). Frequent expression of the multi-drug resistance-associated protein BCRP/MXR/ABCP/ABCG2 in human tumours detected by the BXP-21 monoclonal antibody in paraffin-embedded material. J Pathol.

[B146] Yoh K, Ishii G, Yokose T, Minegishi Y, Tsuta K, Goto K, Nishiwaki Y, Kodama T, Suga M, Ochiai A (2004). Breast cancer resistance protein impacts clinical outcome in platinum-based chemotherapy for advanced non-small cell lung cancer. Clin Cancer Res.

[B147] Jonker JW, Buitelaar M, Wagenaar E, van der Valk MA, Scheffer GL, Scheper RJ, Plosch T, Kuipers F, Elferink RP, Rosing H, Beijnen JH, Schinkel AH (2002). The breast cancer resistance protein protects against a major chlorophyll-derived dietary phototoxin and protoporphyria. Proc Natl Acad Sci U S A.

[B148] Summer R, Kotton DN, Sun X, Ma B, Fitzsimmons K, Fine A (2003). Side population cells and Bcrp1 expression in lung. Am J Physiol Lung Cell Mol Physiol.

[B149] Giangreco A, Shen H, Reynolds SD, Stripp BR (2003). Molecular Phenotype of Airway Side Population Cells. Am J Physiol Lung Cell Mol Physiol.

[B150] Neuringer IP, Randell SH (2004). Stem cells and repair of lung injuries. Respir Res.

[B151] Agassandian M, Mathur SN, Zhou J, Field FJ, Mallampalli RK (2004). Oxysterols trigger ABCA1-mediated basolateral surfactant efflux. Am J Respir Cell Mol Biol.

[B152] Drobnik W, Lindenthal B, Lieser B, Ritter M, Christiansen WT, Liebisch G, Giesa U, Igel M, Borsukova H, Buchler C, Fung-Leung WP, Von Bergmann K, Schmitz G (2001). ATP-binding cassette transporter A1 (ABCA1) affects total body sterol metabolism. Gastroenterology.

[B153] McNeish J, Aiello RJ, Guyot D, Turi T, Gabel C, Aldinger C, Hoppe KL, Roach ML, Royer LJ, de Wet J, Broccardo C, Chimini G, Francone OL (2000). High density lipoprotein deficiency and foam cell accumulation in mice with targeted disruption of ATP-binding cassette transporter-1. Proc Natl Acad Sci U S A.

[B154] Wang N, Lan D, Gerbod-Giannone M, Linsel-Nitschke P, Jehle AW, Chen W, Martinez LO, Tall AR (2003). ATP-binding cassette transporter A7 (ABCA7) binds apolipoprotein A-I and mediates cellular phospholipid but not cholesterol efflux. J Biol Chem.

[B155] Yamano G, Funahashi H, Kawanami O, Zhao LX, Ban N, Uchida Y, Morohoshi T, Ogawa J, Shioda S, Inagaki N (2001). ABCA3 is a lamellar body membrane protein in human lung alveolar type II cells. FEBS Lett.

